# Is EuroSCORE II still a reliable predictor for cardiac surgery mortality in 2022? A retrospective study study

**DOI:** 10.1093/ejcts/ezad294

**Published:** 2023-09-05

**Authors:** Giorgio Mastroiacovo, Alice Bonomi, Monica Ludergnani, Matteo Franchi, Riccardo Maragna, Sergio Pirola, Andrea Baggiano, Alice Caglio, Gianluca Pontone, Gianluca Polvani, Luca Merlino

**Affiliations:** IRCCS Centro Cardiologico Monzino, Department of Cardiovascular Surgery, Milan, Italy; Department of Statistics, IRCCS Centro Cardiologico Monzino, Milan, Italy; Healthcare Management, IRCCS Centro Cardiologico Monzino, Milan, Italy; Department of Statistics, IRCCS Centro Cardiologico Monzino, Milan, Italy; National Centre for Healthcare Research & Pharmacoepidemiology, Head Office at the Department of Statistics and Quantitative Methods, University of Milano-Bicocca, Milan, Italy; Unit of Biostatistics, Epidemiology and Public Health, Department of Statistics and Quantitative Methods, University of Milano-Bicocca, Milan, Italy; Cardiovascular Imaging Department, Centro Cardiologico Monzino IRCCS, 20138, Milan, Italy; IRCCS Centro Cardiologico Monzino, Department of Cardiovascular Surgery, Milan, Italy; Cardiovascular Imaging Department, Centro Cardiologico Monzino IRCCS, 20138, Milan, Italy; Cardiovascular Section, Department of Clinical Sciences and Community Health, University of Milan, 20122, Milan, Italy; Healthcare Management, IRCCS Centro Cardiologico Monzino, Milan, Italy; Cardiovascular Imaging Department, Centro Cardiologico Monzino IRCCS, 20138, Milan, Italy; IRCCS Centro Cardiologico Monzino, Department of Cardiovascular Surgery, Milan, Italy; Department of Surgical and Dental Biomedical Sciences, University of Milan, Italy; Head of Healthcare Management, IRCCS Centro Cardiologico Monzino, Milan, Italy

**Keywords:** EuroSCORE II, cardiac surgery, post-operative mortality, validation study

## Abstract

**OBJECTIVES:**

The European System for Cardiac Operation Risk Evaluation II (EuroSCORE II) is the most common tool used to evaluate the perioperative risk of mortality after cardiac surgery in Europe, and its use is currently recommended by the relevant guidelines. However, recently, its role has been questioned: Several papers have suggested that these algorithms may no longer be adequate for risk prediction due to an overestimation of adult cardiac surgical risk. Our goal was to validate the EuroSCORE II in the prediction of 30-day in-hospital mortality in patients undergoing open cardiac surgery in a high-volume hospital.

**METHODS:**

In this retrospective cohort study, we included all patients who underwent cardiac surgery from January 2016 to May 2022 within the departments of cardiac surgery of the Monzino Cardiology Centre in Milan, Italy. We evaluated the discrimination power of the EuroSCORE II by using the receiver operating characteristic curve and the corresponding area under the curve. We performed calibration plots to assess the concordance between the model’s prediction and the observed outcomes.

**RESULTS:**

A total of 4,034 patients were included (mean age = 65.1 years; 68% males), of which 674 (16.7%) underwent isolated coronary artery bypass grafting. The EuroSCORE II showed a good discrimination power in predicting 30-day in-hospital mortality (area under the curve = 0.834). However, for interventions performed in an elective setting, very low values of the EuroSCORE II overestimated the observed mortality, whereas for interventions performed in an emergency setting, EuroSCORE II values above 10 extensively underestimated the observed mortality.

**CONCLUSIONS:**

Our study suggests that the EuroSCORE II seems not to be a reliable score in estimating the true risk of death, especially in high-risk patients.

**Figure:**
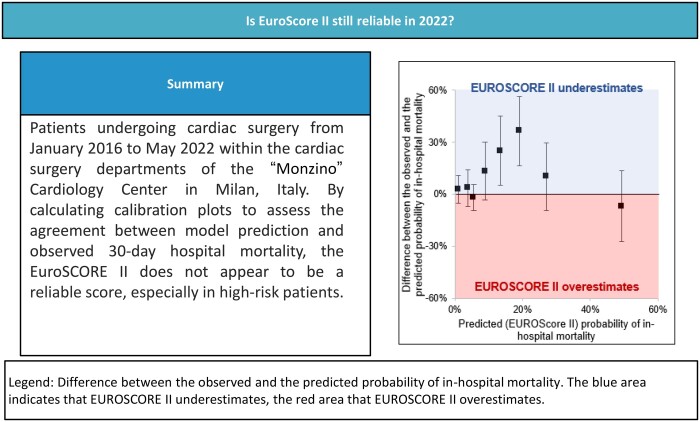

## INTRODUCTION

The European System for Cardiac Operation Risk Evaluation II (EuroSCORE II) is the most common tool used to evaluate the perioperative risk of mortality after cardiac surgery in Europe [1]; indeed, its use is currently recommended by the relevant guidelines [2]. The EuroSCORE II was first published in 2012. Since that time it has been widely used worldwide to predict 30-day mortality in patients undergoing open cardiac surgery. This score was developed by analysing a cohort of more than 22,000 patients who underwent open cardiac surgery in almost 150 hospitals in 43 countries over a 12-week period (May–July 2010) [1]. Several studies confirmed the good performance of the EuroSCORE II in predicting 30-day mortality in patients who underwent major cardiac surgery [3–5]. Due to the composition of the type of surgical interventions performed on the original EuroSCORE II population, mainly coronary artery bypass grafting (CABG), it has up to now shown optimal calibration and discrimination power in isolated CABG [6]. However, recently, its role has been questioned because some evidence has suggested that these algorithms may no longer be adequate for risk prediction due to an overestimation of the adult cardiac surgical risk. Indeed, several studies have shown discrepancies between predicted and observed mortality, particularly in elderly and mid- to high-risk patients [7–9], as well as in valvular surgical subgroups [10–11]. In this study, our goal was to validate the performance of the EuroSCORE II in the prediction of in-hospital mortality in patients undergoing open cardiac surgery (valve, CABG and ascending aorta) in a high-volume hospital in Italy.

## MATERIAL AND METHODS

### Ethics statement

The institutional review board approved the use of the data set for research. The institutional ethical committee approved the study (R 1739/22- CCM 1858), and the requirement for informed written consent was waived on the condition that subjects’ identities were masked.

### Data source and study population

In this retrospective cohort study, we included all the 4,034 patients who underwent cardiac surgery in a 5-year period (January 2016–May 2022) within the department of cardiac surgery of the Monzino Cardiology Centre in Milan, Italy. Patients who had transcatheter/percutaneous valve implants, tumours, congenital heart disease and isolated tricuspid valve surgical procedures were excluded from the study group, because they were not considered in the development of the EuroSCORE II algorithm. Preoperative and demographic information, operative data, perioperative mortality and complications for each included patient were retrieved from the institutional databases of the Monzino Cardiology Centre, which systematically collects data on all patients. Moreover, the calculation of the EuroSCORE II for each patient was made possible by using the information recorded in the hospital databases.

### Outcome

The outcome of interest is the in-hospital mortality that occurred within 30 days from the date of the operation.

### Sample size

Accepting a type I error of 5% and a type II error of 20% and assuming a mortality rate equal to 3.5%, a total 4,034 patients yielded an area under the curve (AUC) of 0.80 with a 95% confidence interval from 0.76 to 0.84.

### Statistical analyses

We used the standardized differences for comparing baseline characteristics of the study cohort and the EuroSCORE population. The normality distribution of the data was evaluated using the Kolmogorov-Smirnov test. According to Austin [12], standardized differences <0.10 were considered negligible. We evaluated the discrimination power of the EuroSCORE by using the receiver operating characteristic curve and the corresponding AUC [13]. We performed calibration plots to assess the concordance between the model’s prediction and the observed outcomes. Predicted versus observed in-hospital mortality probabilities were displayed in a calibration plot. Ideally, the plot should follow a 45-degree line, showing that the predicted risks are equal to the observed outcome frequencies [14]. Analyses were stratified by type of intervention (elective or emergency). Moreover, we evaluated whether the in-hospital mortality was affected by the surgeon performing the intervention. Because our population presented a multilevel structure with patients (level 1) nested within the surgeon (level 2), random effects were included in the logistic models [15]. Three models were fitted: (1) the surgeon random effect alone (null model); (2) the surgeon random effect together with the EuroSCORE; and (3) the surgeon’s expertise (adjusted model). The expertise of the surgeon was defined on the basis of the average number of interventions performed each year, which was then divided into 3 categories: <25, 25 to 90 and ≥90 interventions. In this analysis, surgeons active for less than 1 year were excluded. Third, the surgeon random effect together with the EuroSCORE II, the surgeon’s expertise and the complexity of the intervention (fully adjusted model). The complexity was evaluated separately by 2 different specialists (GM, SP) and classified on a scale from 1 (low complexity) to 5 (high complexity). Within each model, the variance of the distribution of the random effects was used to test the null hypothesis of no surgeon random effect.

## RESULTS

A total of 4,034 patients were included in the cohort. The mean age was 65.1 years and 68% were males. Other baseline characteristics of the study cohort are depicted in Table 1. By comparing the baseline characteristics of the study cohort to those of the original EuroSCORE II population, we found no differences in age, sex and clinical preoperative state between the 2 study populations. In our study group, a significantly lower number of patients with severe renal dysfunction who needed dialysis (standardized difference = −0.14) and with diabetes treated with insulin (standardized difference = −0.10) were reported. The number of in-hospital deaths that occurred within 30 days from the date of the operation was 127 (3.12%).

**Table 1:** Baseline characteristics of the study cohort

EuroSCORE II variables	Study cohort N = 4,034	Percent missing
Age in years—mean (standard deviation)	65.1 (11.5)	0
Sex, male	2,745 (68.0%)	0
COPD	304 (7.5%)	0.7
Diabetes, on insulin	207 (5.1%)	0.7
Peripheral arteriopathy	349 (8.7%)	0
Poor mobility	247 (6.1%)	0
Previous cardiac surgery	437 (10.8%)	0
Active endocarditis	117 (2.9%)	0
Critical preoperative state	148 (3.7%)	0
Neurological dysfunction	100 (2.5%)	0.4
Recent myocardial infarction	199 (4.9%)	0.7
Renal impairment	2,356 (58.4%)	3.6
CC >50–85	1,853 (45.9%)	
CC ≤50	500 (12.4%)	
Dialysis	3 (0.1%)	
NYHA class		2.8
I	776(19.2%)	
II	2,394 (59.4%)	
III	634 (15.7%)	
IV	74 (1.8%)	
CCS IV	43 (1.1%)	1.4
LVEF (%)		3.5
>50	3,430 (85.0%)	
31–50	434 (10.8%)	
21–30	58 (1.4%)	
≤20	10 (0.3%)	
Type of surgery		3.2
Isolated CABG	674 (16.7%)	
Surgery other than isolated CABG	2,155 (53.4%)	
2 procedures	960 (23.8%)	
3 procedures	162 (4.0%)	
Surgery on thoracic aorta	454 (11.3%)	0
Setting		2.8
Election	3595 (89.11%)	
Urgent	200 (5.0%)	
Emergency	126 (3.1%)	
Salvage	7 (0.2%)	

CABG: coronary artery bypass grafting; CC: creatinine clearance; CCS: Canadian Cardiovascular Society; COPD: chronic obstructive pulmonary disease; LVEF: left ventricular ejection fraction; NYHA: New York Heart Association.

Importantly, our population depicts a marked change in the kind of surgical interventions performed most frequently in the last decade. Given that, in 2012, 46.7% of the EuroSCORE II population was represented by patients undergoing isolated CABG, in our population this kind of intervention represents only 16.7% of the total (SD = −0.68) (Table 2).

**Table 2:** Comparison of the baseline characteristics between the study cohort and the EuroSCORE II population

EuroSCORE II Variables	Study cohort N = 4034	EuroSCORE II population N = 22381	Standardized difference
Age in years—mean (standard deviation)	65.1 (11.5)	64.6 (12.5)	0.04
Sex, male	2,745 (68.0%)	15,465 (69.1%)	−0.02
Diabetes, on insulin	207 (5.1%)	1,700 (7.6%)	−0.10
Active endocarditis	117 (2.9%)	492 (2.2%)	0.04
Critical preoperative state	148 (3.7%)	918 (4.1%)	−0.02
Neurological dysfunction	100 (2.5%)	716 (3.2%)	−0.04
Dialysis	3 (0.1%)	246 (1.1%)	−0.14
Isolated CABG	674 (16.7%)	10,452 (46.7%)	−0.68
Surgery on thoracic aorta	454 (11.3%)	1,634 (7.3%)	**0.14**
Urgent	200 (5.0%)	4,140 (18.5%)	**−0.39**

CABG: coronary artery bypass grafting.

**Figure 1:**
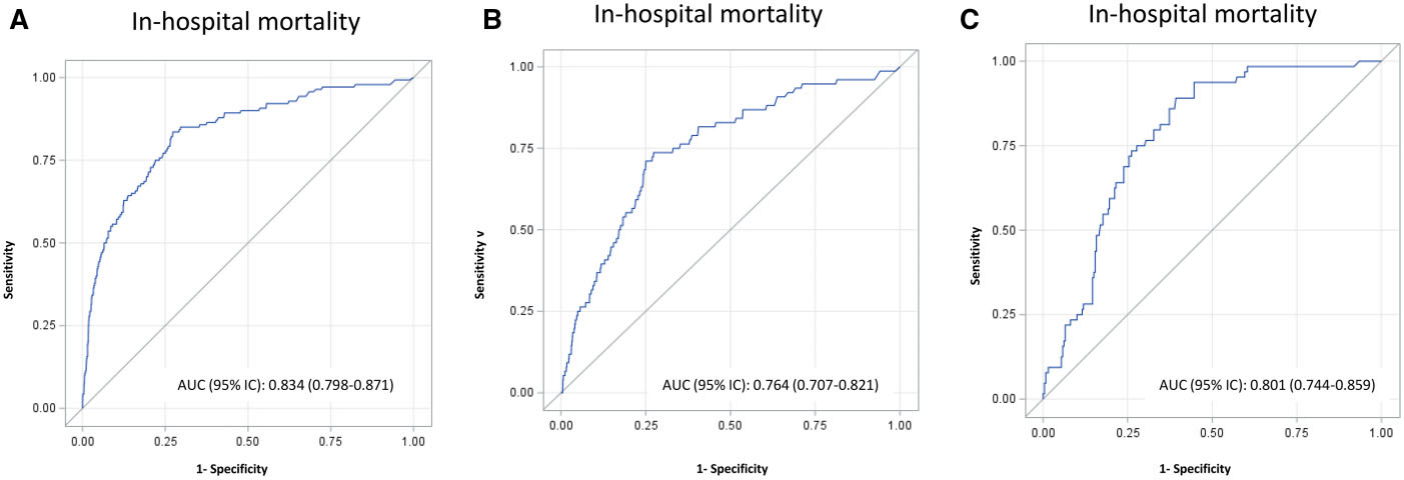
Receiver operating characteristic (ROC) analysis of the EuroSCORE II to predict (**A**) in-hospital mortality in the overall population and according to type of intervention [(**B**) elective or (**C**) emergency-urgent].

**Figure 2:**
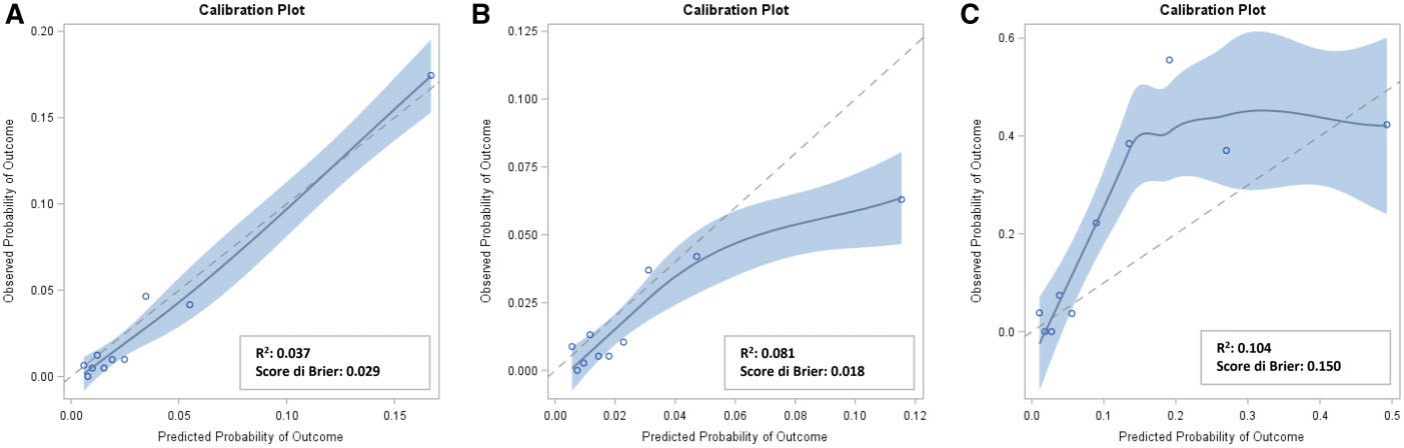
Calibration plots to assess the concordance between predicted (x-axis) and observed (y-axis) in-hospital mortality probabilities in the overall population (**A**) according to type of intervention [elective (**B**) or emergency-urgent (**C**)]. The blue line represents the observed calibration line; the dotted line represents the ideal calibration line.

The EuroSCORE II showed good discrimination power in our population [AUC = 0.834; 95% confidence interval (CI): 0.798–0.871, Fig. 1A). The corresponding figures for patients operated on in elective or in an emergency-urgent setting were 0.764 (95% CI: 0.707–0.821) and 0.801 (95% CI: 0.744–0.859), respectively (Fig. 1B, 1C). Figures 2A and 2B showed the calibration plots that assess the concordance between predicted and observed in-hospital mortality probabilities. Importantly, for interventions performed in an elective setting (Fig. 2A), the very low values of the EuroSCORE II overestimate the real (observed) in-hospital mortality. Moreover, for interventions performed in an emergency-urgent setting (Fig. 2B), EuroSCORE II values above 10 extensively underestimated the observed in-hospital mortality. The same analysis was done by stratifying the population according to CABG, valvular ± CABG and aorta (Supplementary Fig. 2). Results are also confirmed in the subanalysis run in the subgroup of patients undergoing emergency-urgent procedures and EuroSCORE II >10.II >10 (Supplementary Fig. 4).

Finally, the estimated variance of the surgeon-specific random effect was not significant for the completely adjusted model (0.0642; *P* = 0.1788). These findings suggest that the surgeon did not affect the mortality rate, independent of the other covariates.

## DISCUSSION

Risk stratification and risk scoring systems are extremely relevant in adult cardiac surgery because they are needed to guide the decisions of the heart teams by providing an accurate evaluation of patients’ perioperative mortality risks. Risk scores are also important because they allow comparisons of outcomes between different institutions and surgeons, and they permit the evaluation of intrahospital mortality. In recent years, the EuroSCORE II has been widely adopted as a risk prediction tool in adult cardiac surgery, especially in Europe. This model, however, was derived from data collections and from the analysis of 22,381 consecutive cases in 154 European hospitals in 2010, when isolated coronary bypass grafting was by far the most frequent adult cardiac surgical procedure [16]. In the subsequent years, a number of studies sought to validate the performance of the EuroSCORE II in real-world populations of cardiac surgery patients with a variety of ethnic and geographical origins [17–18] and of cardiac pathologies [3–5]. However, several studies showed that the EuroSCORE II does not reliably predict effective mortality after cardiac surgery in different subgroups of patients undergoing cardiac procedures.

In 2012, Biancari *et al.* [19] demonstrated that the EuroSCORE II had a good discrimination power in patients undergoing isolated CABG, but that its ability decreases when applied to valve surgery, especially when it is associated with other procedures [10]. Carino *et al.* [11] in 2021, by analysing a cohort of 2,645 patients who underwent exclusively surgical mitral valve repair, showed that the EuroSCORE II was associated with low discrimination (AUC = 0.68), low accuracy (Brier score = 0.27) and low calibration power with an overestimation of the 30-day mortality in patients affected by degenerative mitral regurgitation.

Barili *et al.* as early as 2012 [20] demonstrated that the EuroSCORE II has an optimal calibration power up to 30% predicted probability, whereas it progressively overpredicts afterwards, suggesting that the EuroSCORE II performs well in the first tertile of risk, but then its ability to discriminate deteriorates dramatically. Similarly, in 2013, Paparella *et al.* [21] demonstrated that the EuroSCORE II model performed well in predicting hospital mortality on a large cohort of patients undergoing cardiac surgery (>6000 procedures; AUC = 0.830). The worst performance was detected for the highest EuroSCORE II deciles, which showed a lower predicted than observed risk of death. Because this risk model was developed 12 years ago, by now, it is expected that it can hardly reflect the current cardiac surgical practice that has changed over the years due to improved surgical techniques and advances in the management of patient postoperatively. Interestingly, 10 years later, our data, collected on 4,034 consecutive patients who underwent cardiac surgery at our centre, confirm the generally good capacity of the EuroSCORE in discriminating patients alive or dead (AUC = 0.834), identifying a value of 10% of predicted mortality above which the EuroSCORE II seems to be unreliable in predicting the mortality of patients undergoing cardiac surgery operations. It is noteworthy that the types of interventions performed on our population of 4,034 patients were consistently different both from those of the EuroSCORE II and of the manuscript from Paparella *et al.* [21], reflecting the extensive evolution of cardiac surgery in the last decade.

Since the EuroSCORE II was published, percutaneous coronary interventions have spread widely as a valid alternative technique to CABG for myocardial revascularization, so that CABG procedures have decreased over the years in several European countries. If patients undergoing procedures of isolated CABG in 2010 and 2013 represented 46.7% and 42% in EuroSCORE II and Paparella *et al.*, respectively, in our population they represent only 16.7%, with most cardiac surgery procedures being represented by valve surgery, which instead accounted for less than 30% of the total procedures on which the EuroSCORE II was estimated. Most importantly, the effect of the surgeon was not considered in the original EuroSCORE study, so we added this further analysis with the goal of evaluating whether the surgeons could play a role in explaining the variability in the observed mortality. Indeed, it may be argued that the difference between observed and predicted probabilities of death shown in the calibration plots in Fig. 2 may be due to the expertise of the surgeons. However, the estimated variance of the surgeon-specific random effect showed that surgeons did not affect patients’ mortality, ruling out the hypothesis that the observed differences can be due to the variability in the expertise of the surgeons.

Our study reflects more accurately the actual cohort of patients who had cardiac surgery than the cohort represented in the EuroSCORE II original publication, showing that, nowadays, this score is not reliable over a value of 10%.

Several limitations should be acknowledged. Because this is a single-centre study, the results may not be generalizable to the clinical practice of different hospital centres; therefore, the percentage of patients undergoing CABG in our centre is lower than that in other hospitals. Because the accuracy of risk prediction of a mortality model depends on the characteristics of the patients being operated on, our findings could be reliably applied only to populations with baseline characteristics similar to the ones analysed in this study. However, the Monzino Cardiology Centre represents a high-volume centre with the highest standard of expertise and competence in cardiac surgery.

## CONCLUSIONS

Our data suggest that, despite a good ability of the EuroSCORE II in discriminating patients alive or dead, it is not reliable in predicting the risk of death, especially when its value is above 10%. New scores are deemed to better evaluate the perioperative mortality risk after cardiac surgery.

## SUPPLEMENTARY MATERIAL

Supplementary material is available at EJCTS online.

## Funding

This study received no specific grant from any funding agency in the public, commercial or not-for-profit sectors.

**Conflict of interest:** None declared

## Supplementary Material

ezad294_Supplementary_Data

## Data Availability

The data underlying this article will be shared on reasonable request to the corresponding author.
