# Supplementary figures and images for: Is EuroSCORE II still a reliable predictor for cardiac surgery mortality in 2022? A retrospective study study
Source: Eur J Cardiothorac Surg. 2023 Sep 5;64(3):ezad294. doi: 10.1093/ejcts/ezad294 (PMC10722878; doi:10.1093/ejcts/ezad294)

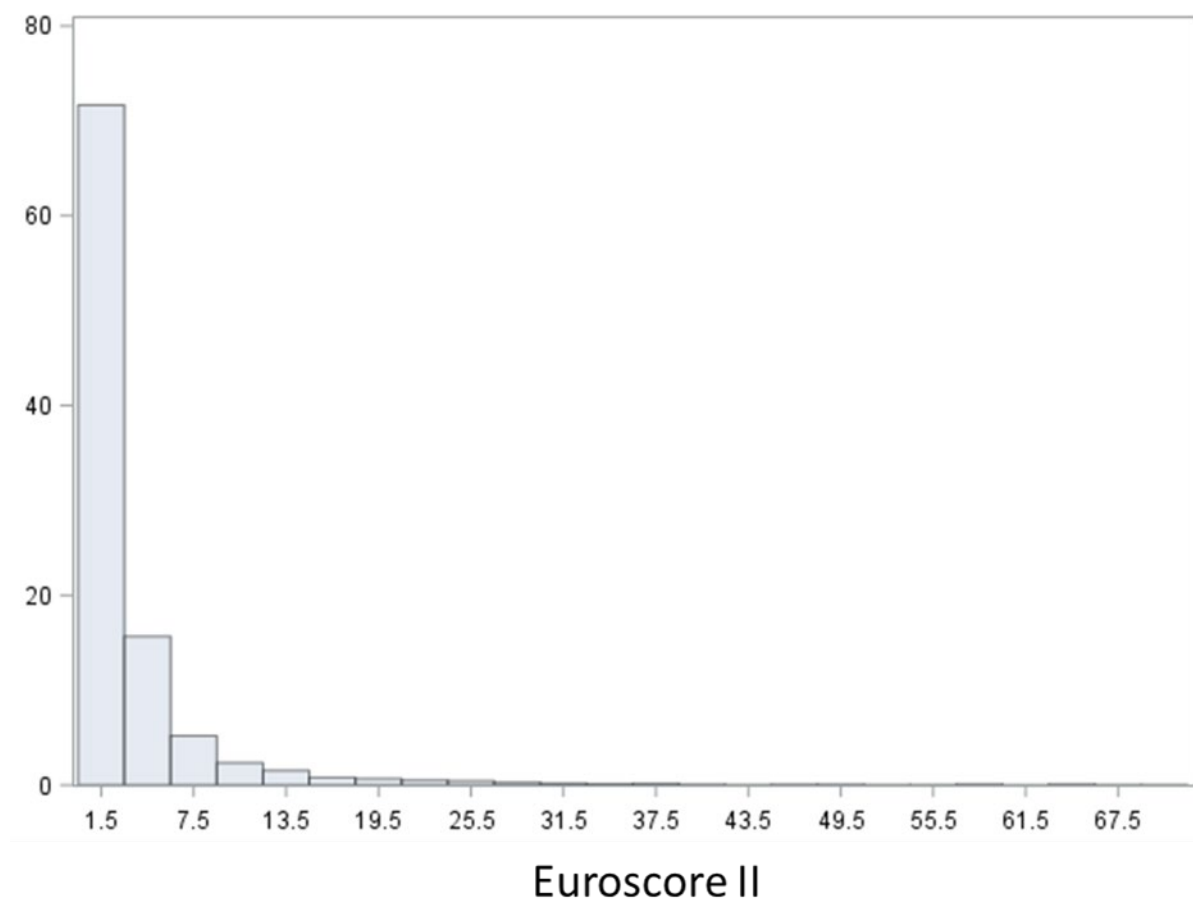

Supplement: ezad294_Supplementary_Data [file ezad294_supplementary_data.zip › Figure 1 Supplementary.pdf]

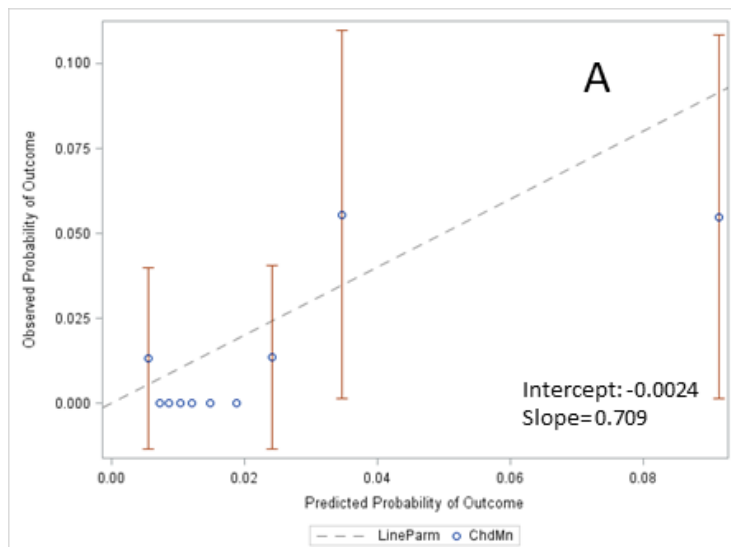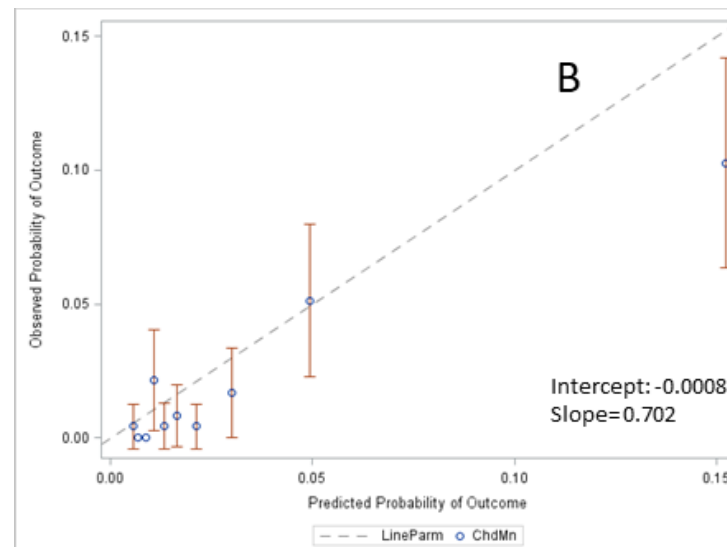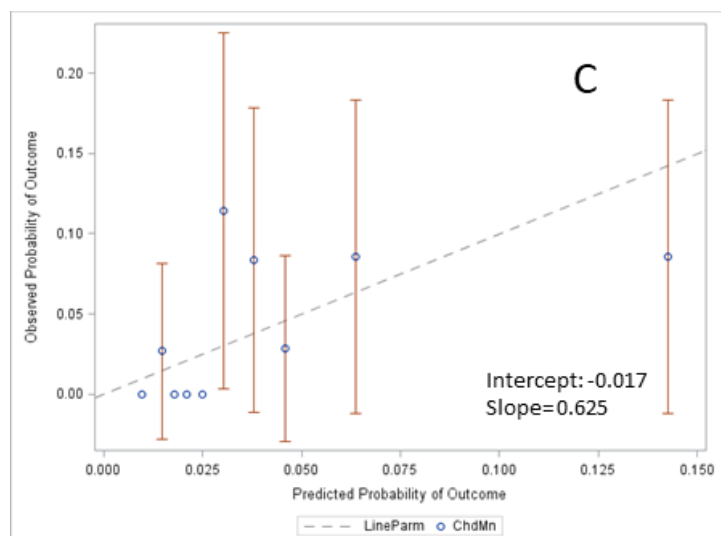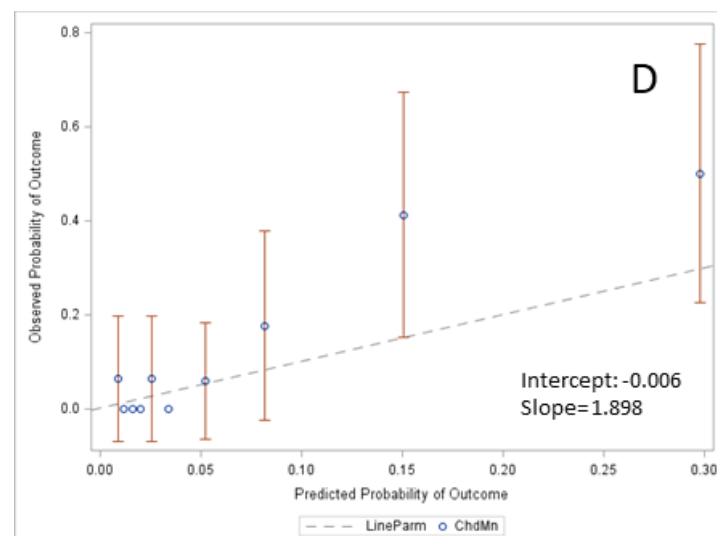

Supplement: ezad294_Supplementary_Data [file ezad294_supplementary_data.zip › Figure 2 Supplementary.pdf]

A

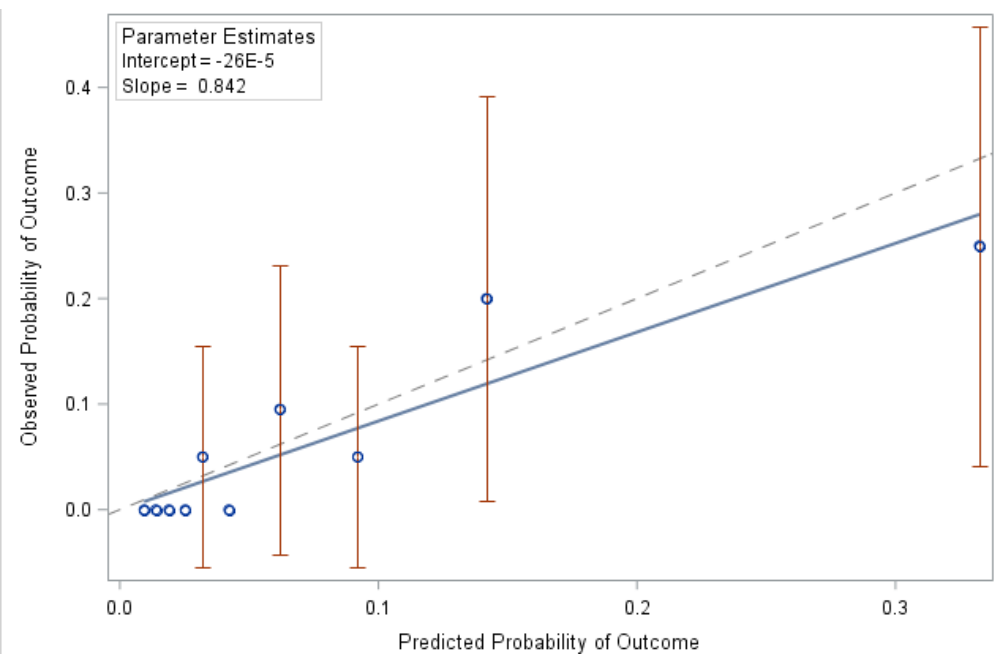

B

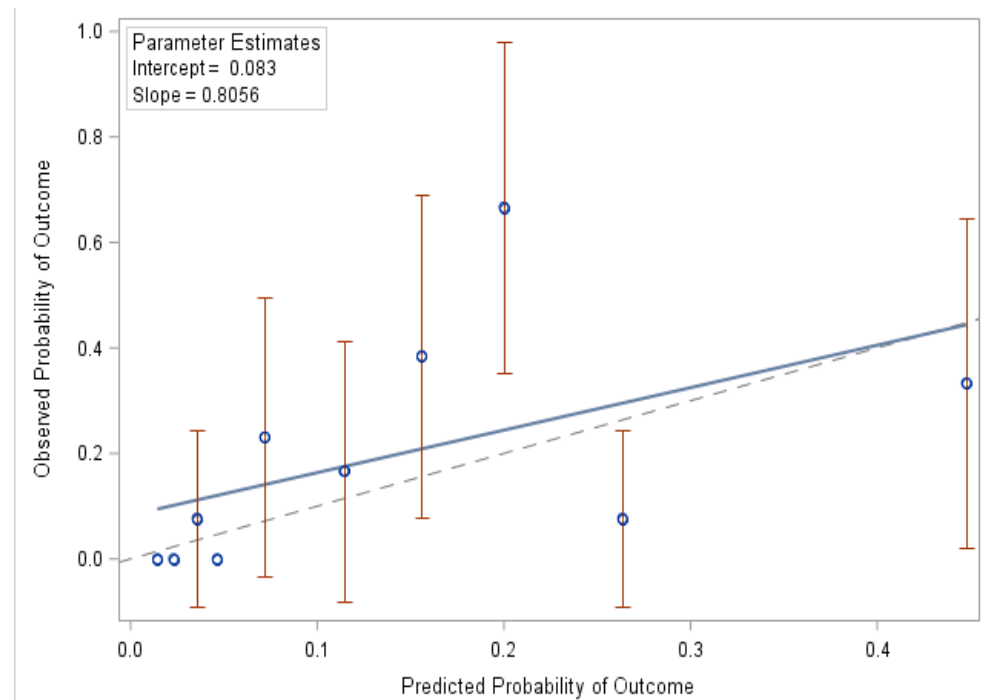

Supplement: ezad294_Supplementary_Data [file ezad294_supplementary_data.zip › Figure 3 Supplementary.pdf]

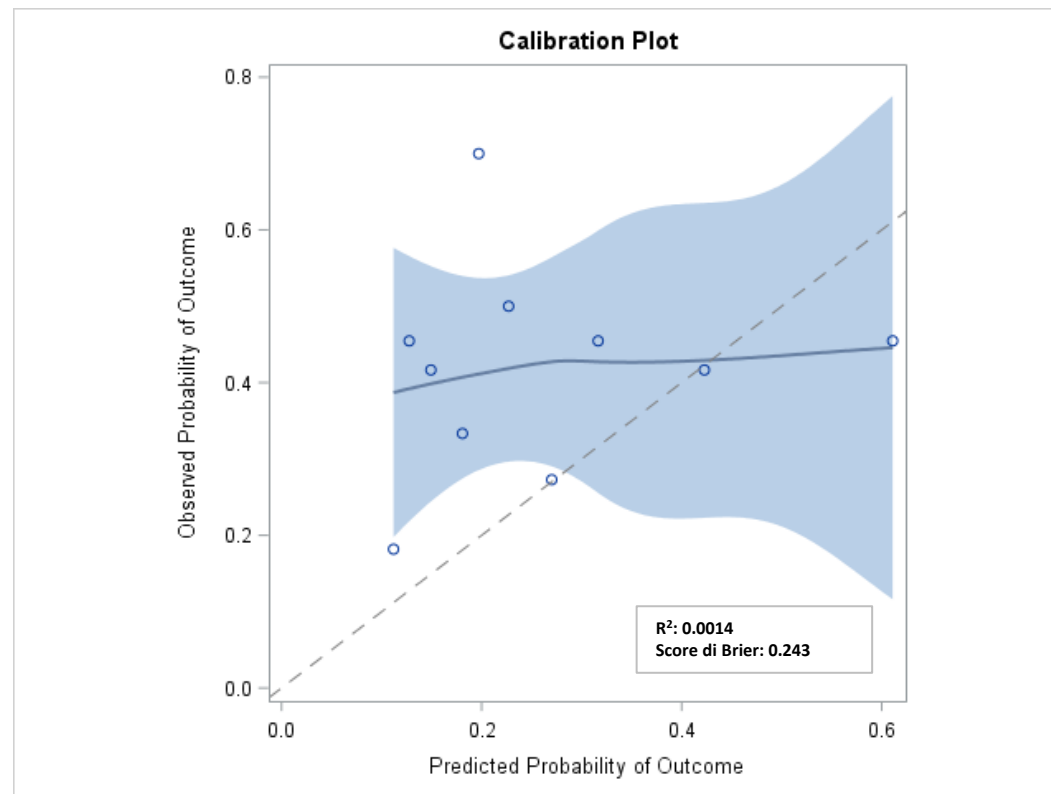

Supplement: ezad294_Supplementary_Data [file ezad294_supplementary_data.zip › Figure 4S.pdf]
